# Chimeric Antisense Oligonucleotide Conjugated to α-Tocopherol

**DOI:** 10.1038/mtna.2014.72

**Published:** 2015-01-13

**Authors:** Tomoko Nishina, Junna Numata, Kazutaka Nishina, Kie Yoshida-Tanaka, Keiko Nitta, Wenying Piao, Rintaro Iwata, Shingo Ito, Hiroya Kuwahara, Takeshi Wada, Hidehiro Mizusawa, Takanori Yokota

**Affiliations:** 1 Department of Neurology and Neurological Science, Graduate School, Tokyo Medical and Dental University, Tokyo, Japan; 2 Core Research for Evolutional Science and Technology (CREST), Japan Science and Technology Agency (JST), Tokyo, Japan; 3 Faculty of Pharmaceutical Sciences, Tokyo University of Science, Chiba, Japan; 4 Department of Pharmaceutical Microbiology, Kumamoto University, Kumamoto, Japan; 5 The Center for Brain Integration Research, Tokyo Medical and Dental University, Tokyo, Japan

## Abstract

We developed an efficient system for delivering short interfering RNA (siRNA) to the liver by using α-tocopherol conjugation. The α-tocopherol–conjugated siRNA was effective and safe for RNA interference–mediated gene silencing *in vivo*. In contrast, when the 13-mer LNA (locked nucleic acid)-DNA gapmer antisense oligonucleotide (ASO) was directly conjugated with α-tocopherol it showed markedly reduced silencing activity in mouse liver. Here, therefore, we tried to extend the 5′-end of the ASO sequence by using 5′-α-tocopherol–conjugated 4- to 7-mers of unlocked nucleic acid (UNA) as a “second wing.” Intravenous injection of mice with this α-tocopherol–conjugated chimeric ASO achieved more potent silencing than ASO alone in the liver, suggesting increased delivery of the ASO to the liver. Within the cells, the UNA wing was cleaved or degraded and α-tocopherol was released from the 13-mer gapmer ASO, resulting in activation of the gapmer. The α-tocopherol–conjugated chimeric ASO showed high efficacy, with hepatic tropism, and was effective and safe for gene silencing *in vivo*. We have thus identified a new, effective LNA-DNA gapmer structure in which drug delivery system (DDS) molecules are bound to ASO with UNA sequences.

## Introduction

Antisense oligonucleotides (ASOs) and small interfering RNA (siRNA) are both recognized therapeutic agents for the silencing of specific genes at the posttranscriptional level.^1^ Chemical modifications, particularly the use of locked nucleic acids (LNAs),^2,3,4^ 2′-*O*-methoxyethyl (2′-*O*-MOE),^5,6^ and constrained ethyl BNA (cEt),^7,8^ markedly improve ASO binding affinity for the target mRNA, resulting in increased steric block efficiency. Currently, the mainstream of the ASO is gapmer ASOs.^1^ Gapmer oligonucleotides, which contain two to five chemically modified nucleotides (LNA, 2′-*O*-MOE RNA, or cEt) as “wings” at each terminus flanking a central 5- to 10-base “gap” of DNA, enable cleavage of the target mRNA by RNase H, which recognizes DNA/RNA heteroduplexes.^9,10^

Recently, the FDA approved Kynamro (mipomersen sodium, Isis Pharmaceuticals, Carlsbad, CA) as a treatment for familial hypercholesterolemia.^11,12^ Kynamro, a DNA 10-mer with 2′-*O*-MOE-modified-5-mers at both ends, targets *Apolipoprotein B* (*ApoB*). It has a strong target gene–silencing effect and greatly reduces serum low-density lipoprotein (LDL)-cholesterol in patients with familial hypercholesteremia. Since the approval of Kynamro, the higher binding affinity of LNAs has prompted the development of far shorter ASOs, which have been shown recently to increase the gene silencing effect, probably because of their increased intracellular availability.^13^ Despite this progress in the design of new chemical modifications of oligonucleotides, methods that improve the potency of oligonucleotide drugs in animals are still highly desirable. The inadequate delivery and poor cellular uptake of oligonucleotides, coupled with their inability to efficiently access the target mRNA during intracellular trafficking,^14^ are major impediments to *in vivo* silencing.^15^

The development of effective delivery systems for oligonucleotides is essential for their clinical application. Previously, we hypothesized that the best *in vivo* carrier for siRNA would be a molecule that the target cells need but cannot synthesize. Vitamins meet these requirements, and the least toxic, fat-soluble vitamin (even at high doses) is vitamin E.^16^ Therefore, we directly conjugated α-tocopherol, a natural isomer of vitamin E, to siRNA and obtained a substantial reduction in the expression of an endogenous gene in mouse liver and brain.^17,18^ In this study, we tried to use α-tocopherol (Toc) conjugation as a delivery system for ASO.

## Results

### Design of Toc-ASO targeting mouse *ApoB* mRNA

**Figure 1:**
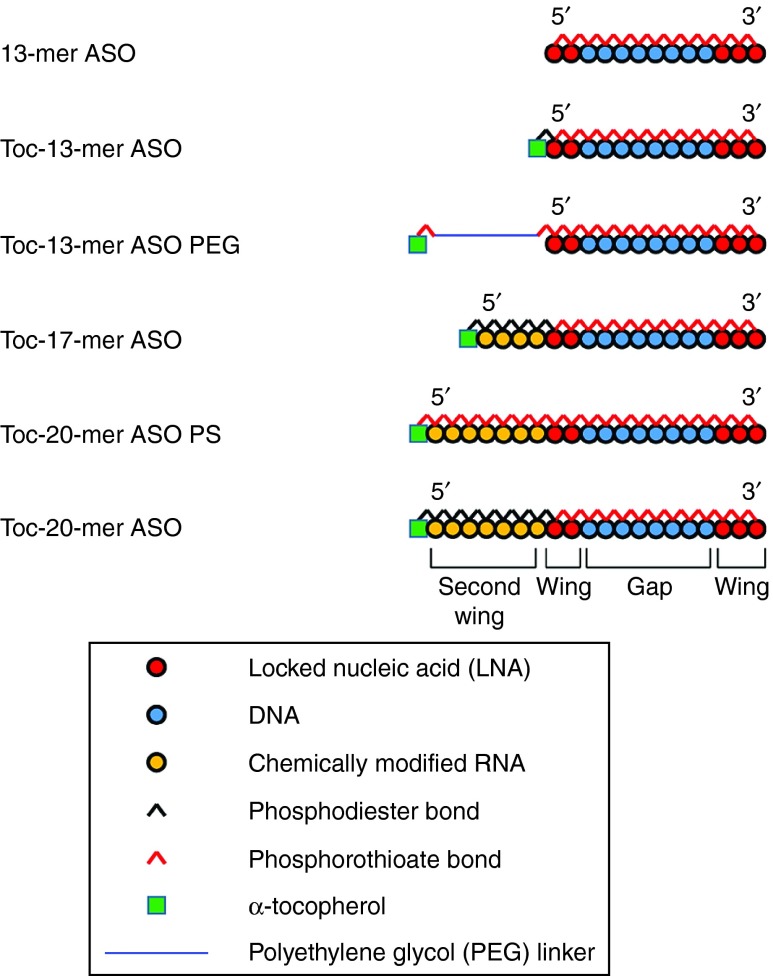
**Design of several types of Toc-ASOs.**

We used the 13-mer LNA/DNA gapmer that targets mouse *ApoB* mRNA (NM_009693) and has been described previously.^13^ Toc was conjugated to several lengths of gapmers or chimeric ASOs. The structures of the α-tocopherol-bound ASO (Toc-ASOs) are shown in **Figure 1**. For example, the 20-mer Toc-ASO is an α-tocopherol–conjugated chimeric 20-mer, with a 7-mer “second wing” (**Figure 1**) of artificial nucleotides extending from the 5′-end of the original 13-mer ASO. The 7-mer second wing was composed of phosphodiester-bound unlocked nucleic acid (UNA) (Toc-20-mer ASO) or phosphorothioate-bound UNA (Toc-20-mer ASO PS). To estimate the effect of the artificial modification second wing, we synthesized α-tocopherol-bound 17-mer ASO with second wing consisting of phosphodiester-bound 2′-Fluoro modified RNA (Toc-17-mer ASO F) and phosphodiester-bound 2′-*O*-methyl RNA (Toc-17-mer ASO OMe).

To estimate the length effect of the second wing, we designed several lengths of Toc-chimeric ASOs that contained phosphodiester-bound UNA, namely Toc-14-mer ASO, Toc-17-mer ASO, and Toc-23-mer ASO. To estimate α-tocopherol conjugation effect, we designed α-tocopherol–unconjugated ASOs with phosphodiester-bound UNA second wing: 14-mer ASO, 17-mer ASO, and 20-mer ASO.

The UV melting temperatures (*T*_m_) of various Toc-ASOs are shown in **Table 1**. All of the Toc-ASOs had approximately the same *T*_m_ value, with the exception of Toc-17-mer ASO OMe and Toc-17-mer ASO F.

### Efficacy of the Toc-ASOs

**Figure 2:**
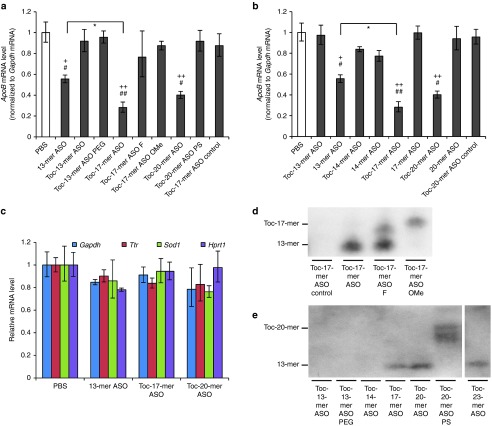
**Gene-silencing effect of intravenous injection of Toc-ASO.** (**a**) Quantitative RT-PCR analyses of *Apolipoprotein B* (*ApoB*) mRNA levels relative to *gapdh* mRNA levels in the liver 3 days after injection of 0.75 mg/kg α-tocopherol–conjugated ASOs. The data shown are relative to those from mice that received PBS alone and are presented as mean values ± SEM (*n* = 3, ^+^*P* < 0.05, ^++^*P* < 0.01 versus PBS, ^#^*P* < 0.05, ^##^*P* < 0.01, versus Toc-17-mer ASO control, and *P < 0.05 Toc-17-mer ASO versus 13-mer ASO). (**b**) Quantitative RT-PCR analyses of *ApoB* mRNA levels (normalized to *gapdh* mRNA levels) in the liver 3 days after injection of 0.75 mg/kg ASOs bound to α-tocopherol by UNA second wings of various lengths or ASO without α-tocopherol. The data shown are relative to those from mice that received PBS alone and are presented as mean values ± SEM (*n* = 3, ^+^*P* < 0.05, ^++^*P* < 0.01 versus PBS, ^#^*P* < 0.05, ^##^*P* < 0.01 versus Toc-20-mer ASO control, and *P < 0.05 between each groups). (**c**) Quantitative RT-PCR analyses of endogenous mRNAs (*Gapdh*, *Ttr*, *Sod1*, and *Hprt*) in the liver 3 days after injection of 0.75 mg/kg ASO, Toc-ASOs, or PBS alone. Data are relative to the total input RNA and are expressed as mean values ± SEM (*n* = 3). (**d**) Northern blot analysis to detect 13-mer gapmer sequences of Toc-ASO in the liver 3 days after injection of mice with 0.75 mg/kg Toc-ASO. (**e**) Northern blot analysis to detect 13-mer gapmer sequences of Toc-ASOs in the liver 3 days after injection of mice with 0.75 mg/kg Toc-ASOs of various lengths.

First, we made a nucleic acid Toc-13-mer ASO, in which the α-tocopherol was directly conjugated to the 13-mer ASO by a phosphodiester bond. Mice were injected with 0.75 mg/kg ASO and examined 3 days later. Quantitative reverse transcriptase polymerase chain reaction (RT-PCR) was performed using total RNA extracted from liver homogenates. We found that the Toc-13-mer ASO had no gene silencing effect (**Figure 2a**). Because conjugation of α-tocopherol interfered with the 13-mer ASO's gene silencing effect, we introduced a spacer between the 13-mer ASO and α-tocopherol. Because Toc-13-mer ASO PEG (α-tocopherol–conjugated to the 13-mer ASO via hexaethylene glycol) also had no effect, we then inserted additional nucleotides as a linker for spacing. Although Toc-20-mer ASO PS had no gene silencing effect (**Figure 2a**), Toc-17-mer and Toc-20-mer ASOs reduced target gene expression, especially Toc-17-mer ASO had significantly greater effect than that of the parent 13-mer ASO (**Figure 2a**).

### Length effect of the second wing

Toc-13-mer (no second wing sequences) and Toc-14-mer ASO had no obvious effect, but Toc-17-mer and Toc-20-mer ASOs decreased the target gene expression. Importantly, these silencing effects were more potent than that of the 13-mer ASO (**Figure 2b**). To verify the advantage of α-tocopherol conjugation, the gene silencing effects of several length of ASOs with α-tocopherol conjugation or without α-tocopherol conjugation were evaluated. The ASOs without α-tocopherol did not have target gene silencing effect (**Figure 2b**). The knockdown effect was specific for the target molecule, as evidenced by the findings that the negative control of Toc-17-mer or Toc-20-mer ASOs targeting an unrelated gene did not affect the *ApoB* mRNA level (**Figure 2a**,**b**), and that *ApoB* targeting Toc-ASOs did not change the levels of the other endogenous mRNAs in the liver—for example, *glyceraldehyde-3-phosphate dehydrogenase* (*Gapdh*), *transthyretin* (*Ttr*), *superoxide dismutase 1* (*Sod1*), and *hypoxanthine guanine phosphoribosyltransferase* (*Hprt*) (**Figure 2c**).

### Chemical modification of the second wing

The target gene silencing effects of Toc-17-mer ASO F and Toc-17-mer ASO OMe was markedly reduced in comparison with Toc-17-mer ASO which had UNA second wing (**Figure 2a**).

To evaluate the difference of mechanisms between effective Toc-ASO and noneffective one, northern blot analysis was performed on mouse liver at 72 hours after 0.75 mg/kg injection of Toc-ASOs. Toc-17-mer ASO OMe produced only one band corresponding to full length of Toc-17-mer ASO OMe itself, and the Toc-17-mer ASO produced a band corresponding to 13-mer ASO, which indicated that the 13-mer ASO was cleaved from Toc-17-mer ASO *in vivo* (**Figure 2d**). Additionally, Toc-17-mer ASO F produced two bands: the cleaved 13-mer and the full length of Toc-17-mer ASO F, it suggested that Toc-17-mer ASO F was thought to be less likely to be cleaved than Toc-17-mer ASO. In the liver samples from the 0.75 mg/kg Toc-ASO–injected mice on 72 hours after injection, the 13-mer band was clearly detected when mice were injected with the Toc-17-mer, Toc-20-mer, and Toc-23-mer ASOs (**Figure 2e**). On the other hand, samples from Toc-ASO–injected mouse liver in which Toc-ASOs had no silencing effect did not produce a 13-mer band (**Figure 2e**).

### Dose dependency and time course of the Toc-ASOs effect

**Figure 3:**
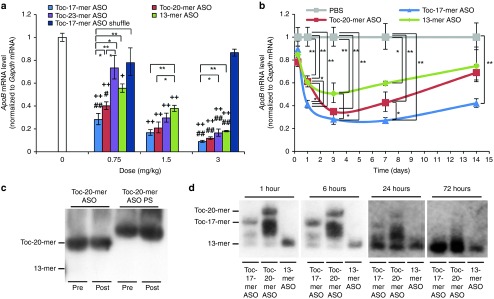
**Dose-dependent reductions in mRNA levels and time course of gene silencing by intravenous injection of Toc-ASOs.** (**a**) Dose-dependent reduction of gene by intravenous injection of Toc-ASOs. Quantitative RT-PCR analyses of *Apolipoprotein B* (*ApoB*) mRNA levels relative to *gapdh* mRNA levels in 3 days after injection of 0.75, 1.5 or 3 mg/kg Toc-ASOs. The data shown are relative to those from mice that received PBS alone and are presented as mean values ± SEM (*n* = 3, ^+^*P* < 0.05, ^++^*P* < 0.01 versus PBS, ^#^*P* < 0.05, ^##^*P* < 0.01 versus Toc-17-mer ASO shuffle, and *P < 0.05, **P < 0.01 between each groups). (**b**) Duration of gene silencing by intravenous injection of Toc-ASOs. Quantitative RT-PCR analyses of *Apolipoprotein B* (*ApoB*) mRNA levels relative to *gapdh* mRNA levels in the liver 6 hours, 1, 3, 7, and 14 after injection of 0.75 mg/kg Toc-ASOs. The data shown are relative to those from mice that received PBS alone and are presented as mean values ± SEM (*n* = 3, *P < 0.05, **P < 0.01). (**c**) The stability of Toc-ASOs. Toc-20-mer ASO and Toc-20-mer ASO PS were incubated in mouse serum with protease inhibitor for 24 hours at 37 °C. The samples were estimated by northern blot analysis to detect 13-mer gapmer sequences of Toc-ASO. (**d**) Northern blot analysis to detect 13-mer gapmer sequences of Toc-ASO in the liver 1 hour to 3 days after injection of mice with 0.75 mg/kg of each ASOs.

We derived dose-response curves from our quantitative RT-PCR results and then calculated the median effective dose (ED_50_)—that is, the dose of ASO that produced a 50% reduction in the target gene expression. We administered 0.75, 1.5, and 3 mg/kg of ASOs to mice and then sampled their livers (**Figure 3a**). We observed a dose dependent gene silencing effect in both 13-mer ASO and Toc-17-mer ASO-injected mice. The respective ED_50_ values for Toc-17-mer ASO, Toc-20-mer ASO, Toc-23-mer ASO, and 13-mer ASO were 24, 60, 145, and 216 nmol/kg. This indicating that Toc-17-mer ASO, Toc-20-mer and Toc-23-mer ASO were more efficacious than 13-mer ASO. We then examined the time courses of their effects. Mice were injected with 0.75 mg/kg of these α-tocopherol–conjugated ASOs, and their livers were collected from 6 hours to 14 days after injection. The gene silencing effects of 13-mer ASO, Toc-17-mer ASO, and Toc-20-mer ASO were observed 1 day after injection. The Toc-17-mer and Toc-20-mer ASOs showed significantly stronger than 13-mer ASO gene silencing effects from days 3 to 14 and days 3 to 7, respectively (**Figure 3b**).

Next, in order to know whether the Toc-ASOs were cleaved before or after reaching the liver, stability studies were performed on Toc-20-mer ASO and Toc-20-mer ASO PS. Both of the Toc-ASOs were incubated in mouse serum with protease inhibitor for 24 hours at 37 °C. In northern blot analysis to detect ASO, both Toc-20-mer-ASO and Toc-20-mer-ASO PS were stable 24 hours after incubation with mice serum (**Figure 3c**). We then examined *in vivo* analysis and performed northern blot analysis to detect ASO. Mice were injected with 0.75 mg/kg of Toc-ASOs, and their livers were collected at 1, 6, 24, and 72 hours after injection. The several bands including the full length of Toc-17-mer and Toc-20-mer ASOs were observed at 24 hours or earlier time point of after injection, and only 13-mer ASOs were detected at 72 hours after injection of Toc-17-mer and Toc-20-mer ASOs (**Figure 3d**). These results suggested that Toc-17-mer ASO and Toc-20-mer ASOs reached the liver with full length, and then were cleaved to 13-mer ASO.

### *In vivo* pharmacokinetics

**Figure 4:**
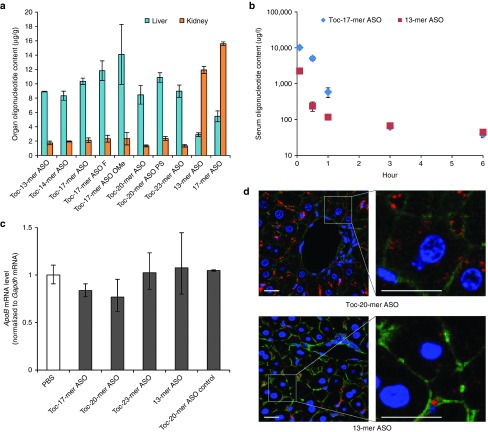
**Biodistribution and pharmacokinesis of Toc-ASO.** (**a**) Measurement of ASO concentrations in each organ 6 hours after injection of 3 mg/kg Alexa Fluor 647–labeled ASOs. The data shown are presented as mean values ± SEM (*n* = 3). The other organs including brain, heart, lung, spleen, intestine and muscle had no signal. (**b**) Measurement of serum ASO concentration 5 minutes to 6 hours after injection of mice with 3 mg/kg of Alexa Fluor 647–labeled ASOs. The data shown are presented as mean values ± SEM (*n* = 3). N.D., not detected. (**c**) Quantitative RT-PCR analyses of *Apolipoprotein B* (*ApoB*) mRNA levels relative to *gapdh* mRNA levels in the intestine 3 days after injection of 3 mg/kg Toc-ASOs. The data shown are relative to those from mice that received PBS alone and are presented as mean values ± SEM (*n* = 3). (**d**) Confocal laser images of mouse liver sections taken 6 hours after injection of 3 mg/kg Alexa Fluor 647–labeled Toc-ASOs. Red, Alexa Fluor 647–labeled ASO; green, Alexa Fluor 488 Phalloidin; blue, Hoechst 33342; Bar = 20 µm.

To determine whether Toc-ASOs was predominantly distributed to liver in mouse after intravenous injection, the *in vivo* tissue accumulation of Toc-ASOs was examined for 6 hours after intravenous injection of Toc-ASOs labeled with Alexa Fluor 647 at the 3′-ends. The accumulation of Toc-ASOs in the mouse liver was ~3.5-fold higher than that of α-tocopherol–unconjugated ASOs while the accumulation of Toc-ASOs in the mouse kidney was approximately sixfold lower than that of α-tocopherol–unconjugated ASOs (**Figure 4a**). No accumulation of Toc-ASOs or α-tocopherol–unconjugated ASOs was observed in brain, heart, lung, spleen, intestine, and muscle because of under detection limit. These results suggest the predominantly delivery of Toc-ASOs to liver after intravenous injection compared to α-tocopherol–unconjugated ASOs.

**Figure 4b** shows the serum concentration-time profiles of Toc-17-mer ASOs and 13-mer ASO after intravenous injection in mice (3 mg/kg). The serum concentration of Toc-17-mer ASOs was greater at 5, 30, and 60 minutes after injection than that of 13-mer ASO in mice while there is no difference at 3 hours after injection between serum concentrations of Toc-17-mer ASOs and 13-mer ASO in mice. As shown in **Table 2**, the area under the serum concentration-time curve (AUC) of Toc-17-mer ASOs was 4.21-fold greater than that of 13-mer ASO in mice. Total body clearance (CLtot), mean residence time (MRT), steady-state volume of distribution (Vdss) and initial elimination rate constant (K_α_) of Toc-17-mer ASOs was lower than that of 13-mer ASO in mice (**Table 2**). There is no significant change of the terminal elimination rate constant (K_β_) between Toc-17-mer ASOs and 13-mer ASO in mice. These results suggested systemic clearance of Toc-17-mer ASO was significantly reduced compared to α-tocopherol–unconjugated ASOs 13-mer ASO, and Toc-17-mer ASO was delivered to liver from the serum more than that of 13-mer ASO in mice (**Table 2**).

Since *ApoB* mRNA was expressed in the intestinal tract, we measured the *ApoB* mRNA silencing effect of Toc-ASOs in intestine. Toc-ASOs had no silencing effect of target gene in intestine even injected 3 mg/kg to the mice (**Figure 4c**). We also examined the delivery to the liver histologically. We found much more intense Alexa Fluor 647 signals in the cytosol of hepatocytes as well as in the sinusoids of mice injected with Toc-20-mer ASO than in those of mice injected with 13-mer ASO (**Figure 4d**).

### Phenotypic analyses of mice using α-tocopherol–conjugated ASOs

**Figure 5:**
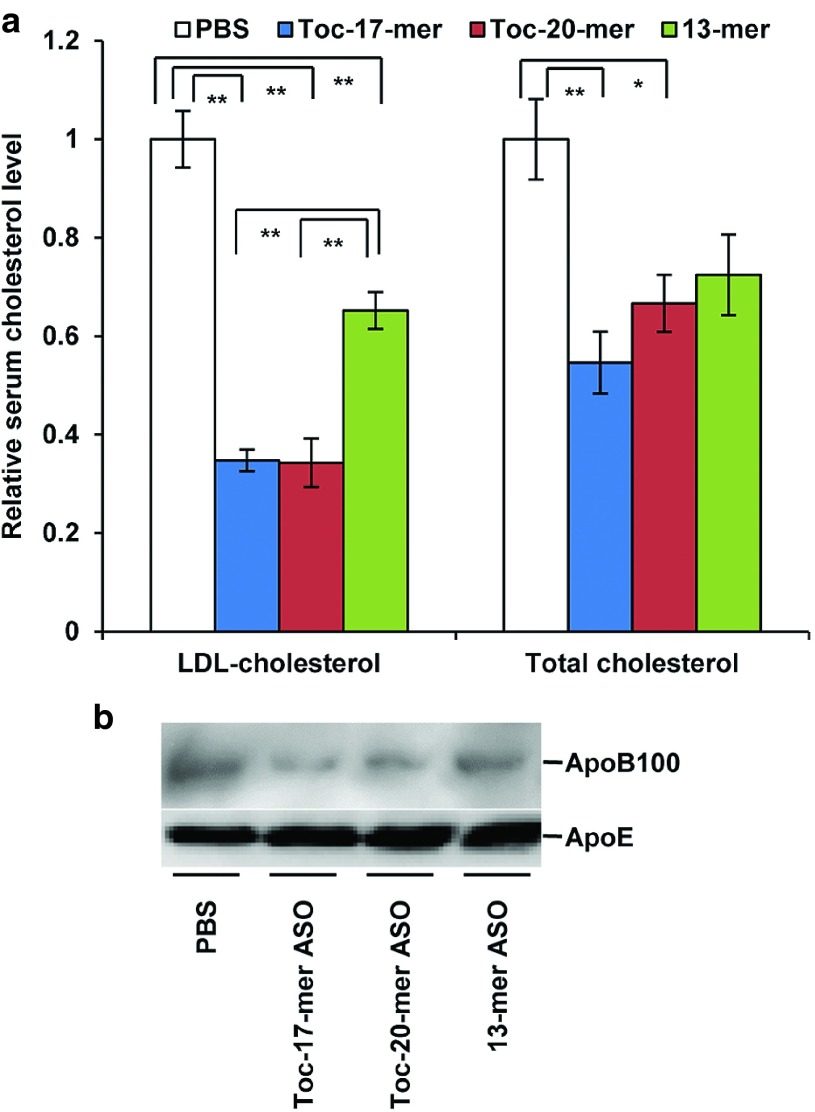
**Phenotypic changes in lipid metabolism caused by inhibition of liver *ApoB* mRNA expression.** (**a**) Decreased levels of serum low density lipoprotein cholesterol (LDL-cholesterol) and total cholesterol after injection of Toc-ASOs. Sera were collected from mice 3 days after the injection of Toc-ASOs. The resultant ratios were normalized against values from mice that were treated with PBS alone. *n* = 3, data shown are mean values ± SEM. *P < 0.05, **P < 0.01 compared with the PBS group. (**b**) Western blot analysis to detect serum ApoB100 proteins in mouse serum 3 days after injection.

The reduction in liver *ApoB* mRNA led to a decrease in serum LDL-cholesterol level. Injection of Toc-17-mer ASO or Toc-20-mer ASO achieved a significant reduction in serum LDL-cholesterol and total cholesterol levels (**Figure 5a**). Western blot analysis of the sera also revealed a clear decrease in ApoB100 content by administration of Toc-17-mer ASO and Toc-20-mer ASO than 13-mer ASO (**Figure 5b**).

### Lack of side effects of α-tocopherol–conjugated ASOs

**Figure 6:**
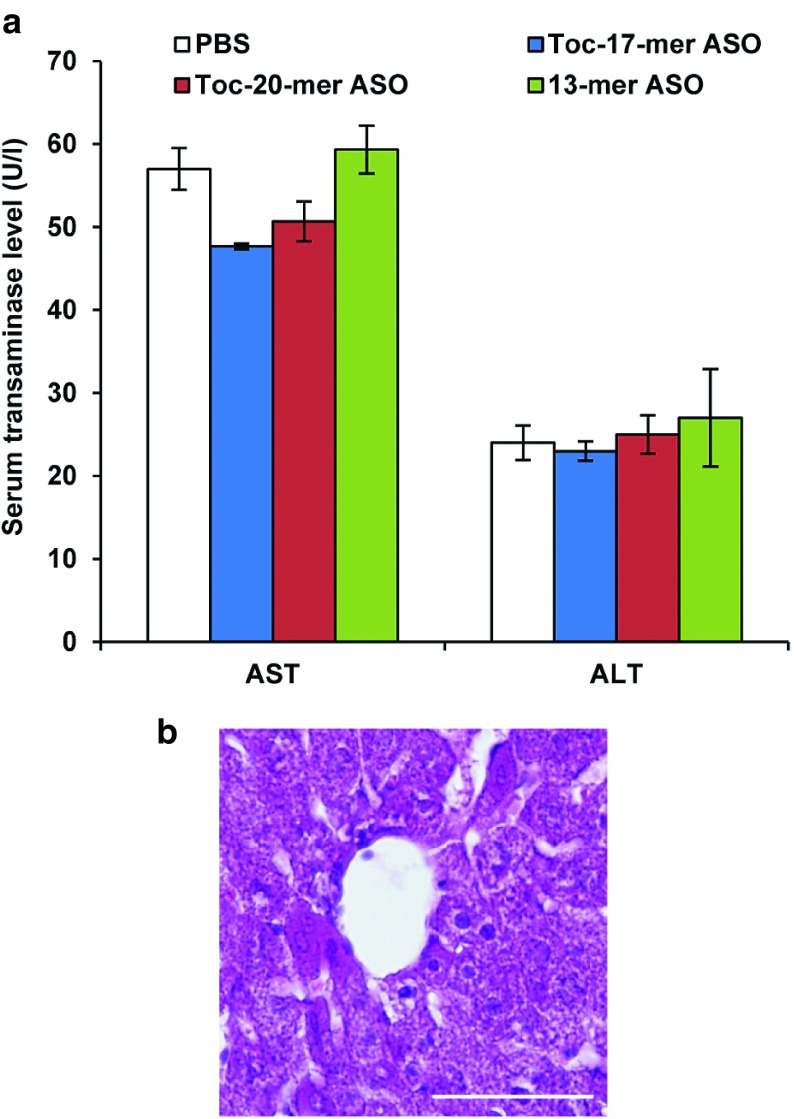
**Evaluation of adverse events.** (**a**) Serum transaminase levels 3 days after injection of 3 mg/kg Toc-ASOs. *n* = 3, data shown are mean values ± SEM. (**b**) Histopathological analyses of liver from Toc-ASO-injected mouse. Liver sections were prepared 3 days after injection of 3 mg/kg Toc-20-mer ASO.

Biochemical analysis of the serum transaminases 3 days after injection of 3 mg/kg ASOs (**Figure 6a**) revealed no marked abnormalities. In addition, no histological abnormalities were found in the livers of 3 mg/kg Toc-17-mer ASO–injected mice (**Figure 6b**).

## Discussion

We previously showed that conjugation of α-tocopherol to siRNA (Toc-siRNA) improves the gene silencing effect of this construct *in vivo*;^17^ however, here, we found that the direct conjugation of α-tocopherol to ASO (Toc-13-mer ASO) abolished this ability (**Figure 2a**). Because we observed more accumulation of Toc-13-mer ASO than of α-tocopherol–unconjugated 13-mer ASO in the liver (**Figure 4a**), we thought that α-tocopherol attenuated the effect of ASO in the hepatocytes. We therefore inserted second wing between the 5′-end of the ASO and the α-tocopherol to avoid α-tocopherol influence. We chose PEG (hexaethylene glycol) or second wings of nucleic acid analogues (*e.g.*, UNA, 2′-F RNA or 2′-*O*-methyl RNA) as linkers. Whereas Toc-13-mer, Toc-13-mer PEG, and Toc-20-mer ASO PS had no effect, inserting the second wing of nucleic acid analogues with a natural phosphodiester internucleotide linkage produced a profound gene slicing effect (**Figure 2a**).

Northern blot analysis of the liver from effective Toc-ASO groups showed 13-mer bands (**Figure 2d**,**e**). Toc-13-mer ASO, Toc-13-mer ASO PEG, and Toc-14-mer ASO could not be observed in northern blot analysis, even though it was certain that the nucleic acids had reached the liver at 6 hour after injection as same amount as Toc-17-mer ASO or Toc-20-mer ASO from the fluorescence measurement (**Figure 4a**). This may have been because the conjugated α-tocopherol inhibited the hybridization of the ASO and the target mRNA when α-tocopherol was too close to the ASO. This clearly indicated that the 13-mer ASO was separated from α-tocopherol by cleavage of the second wing portion, suggesting that silencing of the Toc-ASOs may have been brought about by these cleaved 13-mer ASOs. ASOs bound to α-tocopherol via UNA were not degraded in mice serum (**Figure 3c**). α-tocopherol and second wings of Toc-ASOs were suggested to be cleaved into 13-mer ASO in mice liver (**Figure 3d**). The high effectiveness of Toc-ASOs in liver appears to be dependent on their high-level, *in vivo* delivery to the liver by α-tocopherol and release from α-tocopherol after uptake by the hepatocytes.

Tissue distribution and pharmacokinetic of Toc-17-mer ASO is different from that of 13-mer ASO (**Figure 4a**,**b**). Toc-17-mer ASO was predominantly distributed to liver in mice after intravenous injection. The CLtot of Toc-17-mer ASO was significantly reduced in mice compared to that of 13-mer ASO. These findings suggested that conjugation of α-tocopherol significantly improved the pharmacokinetic profile of ASO.

Toc-13-mer PEG's linker, hexaethylene glycol, was designed to be similar in length to that of the 7-mer oligonucleotide second wing of Toc-20-mer ASO. Both probably uncleaved Toc-13-mer PEG and Toc-20-mer ASO PS (Toc-ASOs with phosphodiesterase-resistant second wings) had no gene-silencing effects, indicating that α-tocopherol conjugation inhibited the effect of the ASO and that merely introducing space between α-tocopherol and the 13-mer ASO does not improve the silencing effect of Toc-ASOs.

Conjugation of α-tocopherol barely changed the *Tm* values of Toc-ASOs (**Table 1**), indicating that α-tocopherol conjugation did not markedly affect the duplex formation of ASO with the target mRNA *in vitro*. However, *in vivo*, the high hydrophobicity of α-tocopherol may have impeded gene silencing by interfering with the Toc-ASO's access to the target RNA during its intracellular trafficking by either binding to the membranes of intracellular organelles or intracellular proteins or inhibiting the function of proteins necessary for gene expression (including RNase H), or both.

Toc-17-mer ASO with the UNA second wing was more effective than Toc-17-mer ASO OMe or Toc-17-mer ASO F (**Figure 2a**). Since the arrival amounts to the liver of Toc-17-mer ASO, Toc-17-mer ASO OMe, or Toc-17-mer ASO F were not different so much (**Figure 4a**), the difference of the effects are not considered to be due to the quantity in the liver. Northern blot analysis showed a band signal for the 13-mer ASO in the sample containing the Toc-17-mer ASO with the UNA second wing (**Figure 2d**), and it suggested that the UNA second wing with phosphorothioate bond was more easily cleaved than the 2′-*O*-Me or the 2′-F second wing in hepatocytes.

When we consider the relationship between the efficiency of the Toc-ASO with the phosphate-bound UNA second wing and the length of the second wing, we see that the efficiency was highest for the Toc-17-mer ASO and attenuated for the Toc-ASO with the longer second wing. In contrast, the Toc-14-mer ASO was not effective (**Figure 2b**). Given that we did not observe a 13-mer band in the northern blot with the Toc-14-mer ASO (**Figure 2d**), it may be that the Toc-14-mer ASO's α-tocopherol was not cleaved from the ASO. Therefore, a single UNA may not be enough to be recognized by nucleases. We thought of three possible reasons why the longer second wing attenuated the Toc-ASO's efficacy: (i) it took more time to cleave the longer second wing and (ii) the longer second wing became an obstacle for recognition by the phosphodiesterase.

The RNase H cleavage–mediated silencing mechanism of the gapmer ASO dramatically improved the effectiveness of the ASO. The nucleotide analogues that served as wings (**Figure 1**), such as LNA, MOE, or cET, have been investigated to further increase the effectiveness of the gapmer. Optimization of gapmers (the length of the gap and the wings) has been shown to increase their effectiveness; the wing-gap-wing gapmer nucleotide composition of 2-8-3 or 2-8-2 in the LNA,^13^ 5-10-5 (Mipomersen) in MOE,^12^ and 3-10-3 in cET^19^ were all reported to be excellent. We used the 2-8-3 13-mer ASO, which was one of the most effective LNA-containing gapmers. However, because the amount of unconjugated 13-mer ASO that reached the kidney was higher than that reaching the liver, the 13-mer ASO could not fully exert a sufficient effect in the liver, the target organ (**Figure 4a**). Here, we succeeded in improving the amount of ASO delivered to the liver by binding a delivery molecule to the ASO.

There have been several recent reports of organ-specific delivery of molecules conjugated to siRNA, including cholesterol to the liver^20^ or brain capillary endothelial cells,^21^ GalNac to the liver,^22^ atelocollagen to the liver,^23^ dynamic polyconjugates to the liver,^24^ peptide derived from rabies virus glycoprotein to neurons,^25^ and oligo-9-arginine peptide to T cells,^26^ as well as the delivery of peptide conjugated to phosphorodiamidate morpholino oligomers to skeletal muscle.^27^ Recently, Prakash *et al.* reported GalNac conjugated to ASO with linker, and the GalNac-conjugated ASO improved potency in mouse liver resulted in enhanced ASO delivery to hepatocytes.^28^ The GalNac-conjugated ASO is metabolized to liberate the parent ASO in the liver,^28^ similar to Toc-ASO.

Our chimeric ASO with the appropriately cleavable second wing can be applied to different organs or cells by selecting different delivery molecules. Further improvements of the molecule design of the second wing will help further potency and safety for the clinical application of this new type of chimeric oligonucleotide.

## Materials and methods

*Design and synthesis of ASOs.* A series of DNA-LNA gapmers of different lengths (13- to 23-mers) were designed to target mouse *ApoB* mRNA (NM_009693).^13^ The ASOs were synthesized by Gene Design (Osaka, Japan). The sequences of the ASOs targeting *ApoB* mRNA were as follows: 13-mer ASO, 5′-G*C*a*t*t*g*g*t*a*t*T*C*A-3′; Toc-13-mer ASO PEG, 5′-X*PEG*G*C*a*t*t*g*g*t*a*t*T*C*A-3′; Toc-13-mer ASO, 5′-**X**G*C*a*t*t*g*g*t*a*t*T*C*A-3′; 14-mer ASO, 5′-*A*G*C*a*t*t*g*g*t*a*t*T*C*A-3′; Toc-14-mer ASO, 5′-**X***A*G*C*a*t*t*g*g*t*a*t*T*C*A-3′; 17-mer ASO, 5′-*UCCA*G*C*a*t*t*g*g*t*a*t*T*C*A-3′; Toc-17-mer ASO, 5′-**X***UCCA*G*C*a*t*t*g*g*t*a*t*T*C*A-3′; Toc-17-mer ASO F, 5′-**X***UCCA*G*C*a*t*t*g*g*t*a*t*T*C*A-3′; Toc-17-mer ASO OMe, 5′-**X**UCCAG*C*a*t*t*g*g*t*a*t*T*C*A-3′; Toc -20-mer ASO PS, 5′-**X**A*A*G*U*C*C*A*G*C*a*t*t*g*g*t*a*t*T*C*A-3′; 20-mer ASO, 5′-*AAGUCCA*G*C*a*t*t*g*g*t*a*t*T*C*A-3′; Toc-20-mer ASO, 5′-**X***AAGUCCA*G*C*a*t*t*g*g*t*a*t*T*C*A-3′; and Toc-23-mer ASO, 5′-**X***AUAAAGUCCA*G*C*a*t*t*g*g*t*a*t*T*C*A-3′. The shuffle sequence of the ASO targeting *ApoB* mRNA was as follows: Toc-17-mer ASO shuffle, 5′-**X***UCCA*C*G*a*t*t*g*g*t*a*t*C*G*C; The sequence of the ASO targeting human *TTR* mRNA (NM_000371) was as follows: Toc-20-mer ASO control, 5′-**X***TGTTTTA*T*G*t*c*t*c*t*g*c*c*T*G*G-3′; The sequence of the ASO targeting SRB1 mRNA (NM_000371) was as follows: Toc-17-mer ASO control, 5′-**X***GCUU*C*A*g*t*c*a*t*g*a*c*T*T*C-3′; where the asterisks represent phosphorothioate linkages, the upper case boldface letter X represents α-tocopherol, the upper case italicized letters represent UNA, the lower case letters represent DNA, the underlined characters represent 2′-*O*-methyl sugar modification, the underlined upper case italicized letters represent 2′-Fluoro modification, and the upper case letters represent LNA (capital C denotes LNA methylcytosine). Alexa Fluor 647 fluorophores were covalently bound to the 3′-ends of the ASOs, and α-tocopherol was covalently bound to the 5′-ends of the ASOs.

*UV melting analyses.* UV absorbance versus temperature profile measurements were performed with an eight-sample cell changer, in quartz cells of 1-cm path length. The variations with temperature in the differences in UV absorbance measured at wavelengths of 260 nm and 320 nm were monitored. The samples containing the oligonucleotides with the complementary RNA, 5′-ugaauaccaaugcuggacuuuauaaccaatc-3′, (1.25 μmol/l in PBS) were first rapidly heated to 90 °C, maintained at 90 °C for 10 minutes, and then allowed to cool to 0 °C at a rate of 0.5 °C/minute. These samples were then left at 0 °C for 30 minutes, and the dissociation was recorded by heating to 90 °C at a rate of 0.5 °C/minute.

*Mouse studies.* Wild type Crlj:CD1 (ICR) mice or C57BL/6 mice aged 4–5 weeks (Oriental Yeast, Tokyo, Japan) were kept on a 12-hour light/dark cycle in a pathogen-free animal facility with free access to food and water. ASOs were administered to the mice via tail vein injection based upon body weight (0.75–6 mg/kg). All oligonucleotides were formulated in PBS, which also served as the control. The oligonucleotides were administered via either a single injection or repeated injections. All animal experiments were performed with more than three mice, and all procedures were carried out according to Tokyo Medical and Dental University's ethical and safety guidelines for animal experiments (#0140144A). Sera were collected 3 days after the final injection to measure LDL-cholesterol levels and for western blot analysis. For postmortem analyses, mice were deeply anesthetized with intraperitoneally administered 60 mg/kg pentobarbital and then sacrificed by transcardiac perfusion with PBS after confirming the absence of the blink reflex.

*Quantitative real-time polymerase chain reaction.* Total RNA was extracted from mouse liver or intestine by using Isogen (Nippon Gene, Tokyo, Japan). To detect mRNA, DNase-treated RNA (2 μg) was reverse-transcribed with SuperScript III and Random Hexamers (Life Technologies, Carlsbad, CA). To detect short RNAs, including DNA-LNA gapmer, quantitative RT-PCR analysis was performed by using a TaqMan MicroRNA Reverse Transcription Kit (Applied Biosystems, Foster City, CA) and a Light Cycler 480 Real-Time PCR Instrument (Roche Diagnostics, Mannheim, Germany). The primers and probes for the DNA/LNA gapmers and mouse *ApoB*, *Gapdh* (NM_008084), *Ttr* (NM_013697), *Sod1* (NM_011434), and *Hprt* (NM_013556) genes were designed by Applied Biosystems.

*Isolation of the lipoprotein fraction from serum.* The LDL fraction was prepared by ultracentrifugation according to a previously published method,^21^ with modification. First, a half-volume of a solution of density 1.182 g/ml was layered onto one volume of mouse serum and centrifuged for 3.6 hours at 337,000*g* at 16 °C. The half-volume of the upper solution was set aside for use in experiments as the LDL fraction.

*Western blot analysis.* The LDL fraction from mouse serum samples (2 μl) was diluted with 18 μl of PBS, mixed with 5 μl of Laemmli sample buffer (Bio-Rad, Hercules, CA), and then denatured at 95 °C for 2 minutes. Total proteins were separated by electrophoresis on a 5–20% gradient polyacrylamide gel (ATTO Corporation, Tokyo, Japan) and transferred onto polyvinylidene difluoride membranes. Blots were probed with goat primary antibodies against ApoE (1:500, sc-6384, Santa Cruz Biotechnology, Santa Cruz, CA) and ApoB (1:500, sc-11795, Santa Cruz Biotechnology), and then incubated with an anti-goat secondary antibody (1:2,000, sc-2020, Santa Cruz Biotechnology) conjugated with horseradish peroxidase. Blots were visualized with SuperSignal West Femto Maximum Sensitivity Substrate (Thermo Fisher Scientific, Waltham, MA) and analyzed by use of a ChemiDoc System (Bio-Rad).

*Northern blot analysis.* Total RNA was extracted from mouse liver by using Isogen II (Nippon Gene). Total RNA (30 μg) was separated by electrophoresis through an 18% polyacrylamide-urea gel and transferred to a Hybond-N^+^ membrane (Amersham Biosciences, Piscataway, NJ). The blot was hybridized with a probe corresponding to the ASO sequence. The sequence of the probe for detecting ASO was 5′-TGAataccaatGC-3′; the lower case letters represent DNA, and the upper case letters represent LNA (capital C denotes LNA methylcytosine). The digoxigenin-ddUTP was covalently bound to the 5′-end of the ASO probe. The signals were visualized with a Gene Images CDP-star Detection Kit (Amersham Biosciences).

*Evaluation of blood chemistry.* A single 0.75 mg/kg dose of ASOs in PBS was injected into the tail vein of mice. Sera were collected 3 days after injection, and blood chemistry was assessed.

*Nuclease stability assays.* Nuclease stability assays were performed according to a previously published method^29^ with a modification. Briefly, ASOs were incubated in mouse serum with protease inhibitor for 24 hours at 37 °C. RNA was extracted using Isogen II, and were examined by northern blot analysis.

*Measurement of ASO concentration in each organ.* Mice were injected with Alexa Fluor 647–labeled ASOs; 6 hours later, tissues were obtained from various organs (brain, heart, lung, liver, kidney, spleen, intestine, and muscle). Tissues were homogenized in 500 μl of phosphate-buffered saline (PBS, Sigma-Aldrich, St Louis, MO). The concentration of Alexa Fluor 647 was measured by using i-control (Tecan, Männedorf, Switzerland).

*Plasma pharmacokinetic studies.* Each mouse received a bolus intravenous injection of Alexa Fluor 647–labeled Toc-17-mer ASO or Alexa Fluor 647–labeled 13-mer ASOs into tail vein. Blood samples were collected at indicated times (5, 30, 60, 180, and 360 minutes). The serum concentration of Alexa Fluor 647 was measured by using i-control (Tecan). The plasma concentration versus time data were analyzed by MOMENT based on the model-independent moment analysis method.^30^ The nonlinear least-squares regression analysis program MULTI.^31^ The pharmacokinetics parameters such as area under the serum concentration-time curve (AUC), the total body clearance (CLtot), the mean residence time (MRT) and the steady-state volume of distribution (Vdss), elimination rate constants (K_α_ and K_β_) were calculated as described previously.^32^

*Histopathological analyses.* For pathological analyses, mouse liver was collected 3 days after injection and then postfixed in 4% paraformaldehyde in PBS for 6 hours, embedded in paraffin, cut into 4-μm thick sections with a Leica CM 3050 S cryostat (Leica Microsystems, Wetzlar, Germany), and stained with hematoxylin and eosin (Muto Pure Chemicals, Tokyo, Japan). The slides were analyzed under an Olympus AX80 Automatic Research Photomicroscope (Olympus, Tokyo, Japan). To analyze the distribution of ASO in the liver, 0.75 mg/kg Alexa Fluor 647–labeled ASOs in PBS was injected into mouse tail veins. Mouse liver was collected 3 days after injection, fixed in 4% paraformaldehyde in PBS for 12 hours, and then snap-frozen in liquid nitrogen. Tissue sections (10 μm) were prepared with a Leica CM3050 S cryostat (Leica Microsystems). The sections were stained with Hoechst 33342 (Sigma-Aldrich) to visualize nuclei and with 13 nmol/l Alexa Fluor 488 phalloidin (Life Technologies) to visualize cell membranes. They were then analyzed under a LSM 510 confocal microscope (Carl Zeiss MicroImaging GmbH).

*Statistical analysis.* All data represent means ± SEM. Student's two-tailed *t*-tests were used to determine the significance of differences between two groups in quantitative RT-PCR assays, analyses of lipoprotein levels in serum and plasma pharmacokinetic studies. One-way ANOVA followed by Tukey's test were used for multiple comparisons between pairs of groups.

## Figures and Tables

**Table 1:**
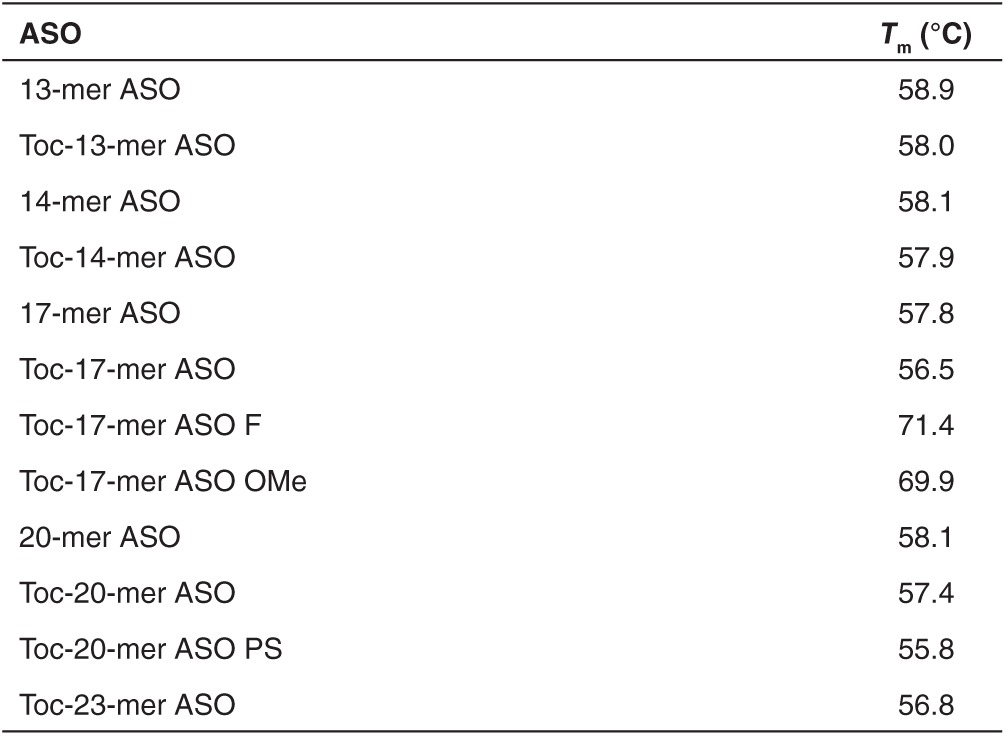
Melting temperatures (*T*_m_) of ASOs targeting mouse *Apolipoprotein B* (*ApoB*) mRNA

**Table 2:**
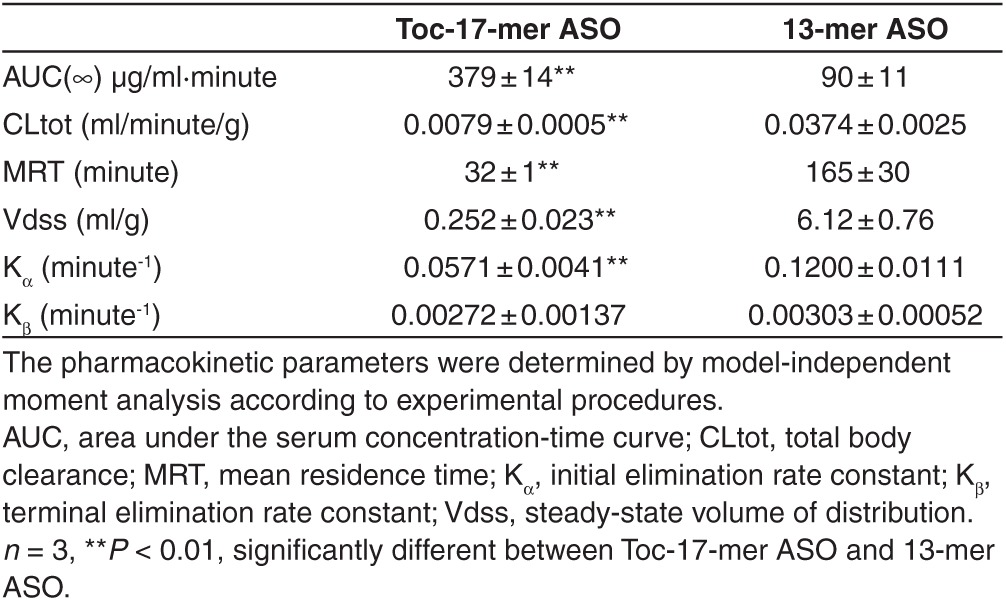
Pharmacokinetic parameters of Toc-17-mer ASO and 13-mer ASO after 3 mg/kg intravenous administration
